# Comorbidities and Pregnancy Do Not Affect Local Recurrence in Patients With Giant Cell Tumour of Bone

**DOI:** 10.7759/cureus.9164

**Published:** 2020-07-13

**Authors:** Emma L Howard, Jonathan Gregory, Kim Tsoi, Scott Evans, Adrienne Flanagan, Paul Cool

**Affiliations:** 1 Orthopaedic Oncology, Robert Jones and Agnes Hunt Orthopaedic Hospital NHS Foundation Trust, Oswestry, GBR; 2 Orthopaedic Oncology, The Royal Orthopaedic Hospital NHS Foundation Trust, Birmingham, GBR; 3 Pathology, The Royal Orthopaedic Hospital NHS Foundation Trust, Stanmore, GBR; 4 Medical Sciences, Keele University, Keele, GBR

**Keywords:** giant cell tumor of bone, epidemiology, physiopathology, pregnancy, complications, neoplastic, autoimmune diseases, treatment outcome, giant cell tumour of bone

## Abstract

This study evaluates the relationship between pregnancy, comorbid conditions and giant cell tumour of bone. Furthermore, it examines if pregnancy and comorbid conditions affect the outcome following treatment for this tumour.

A multi-centre retrospective review was conducted of consecutive patients with a confirmed histological diagnosis of giant cell tumour of bone between June 2012 and May 2017. A total of 195 patients were identified from two centres. Of these, 168 patients were treated with curative intent and had more than six months follow-up. Data were collected on pregnancy status, comorbid conditions, site of disease, surgical management and local recurrence rates. Statistical analysis included the Fisher exact test and Kaplan-Meier survival analysis.

There were 72 females of childbearing age, of which 15 (21%) were currently pregnant or had been pregnant within the last six months. The pregnancy rate is higher than the highest reported pregnancy rate over the last 10 years (8.4%; Fisher test, p = 0.033). Women were more likely to have a comorbid condition than men (Fisher test, p < 0.002) and had a higher rate of autoimmune disease than the normal population (p = 0.015). Men were older than women (Wilcoxon test, p = 0.046) and had less risk of local recurrence (logrank test, p = 0.014). Pregnancy or comorbid conditions did not increase the local recurrence rate. Predictors for local recurrence included location in the distal radius (logrank test, p < 0.001), intralesional treatment (logrank test, p = 0.008) and age less than 40 (logrank test, p = 0.043).

In conclusion, giant cell tumour of bone is more common in pregnant females and patients with immune disease. Comorbidities and pregnancy do not affect the local recurrence rate. Male patients over 40 years of age have a lower risk of local recurrence, and patients with disease in the distal radius have a high risk of recurrence.

## Introduction

Giant cell tumours of bone (GCTB) are aggressive, locally destructive, benign bone tumours. They typically present in the third and fourth decades of life and account for approximately 5% of primary bone tumours [1]. Females are more commonly affected, with a reported predilection of 1.5:1 [2]. There are several clinical characteristics and presentations of GCTB that are still poorly understood.

GCTB presenting in the perinatal period, although rare, has been reported since the 1950s [3]. To date, there is no study investigating the relationship between perinatal GCTB and local recurrence rates.

Empirically, the authors noted that male patients presenting with GCTB were usually fit and healthy, whereas female patients often had other comorbidities, many of which were autoimmune in origin [4].

The aim of this paper was to investigate the relationship between pregnancy, comorbid conditions and GCTB, and whether these clinical presentations affect local recurrence rates. Previously described risk factors for local recurrence, including surgical management and site of disease, were also investigated.

## Materials and methods

A retrospective review was conducted in two UK primary bone tumour centres of consecutive patients with a confirmed histological diagnosis of GCTB between June 2012 and May 2017. Both institutions operate a biobank approved by the Health Research Authority that includes informed consent. Patients with malignant GCTB were excluded from this review. Local recurrence was evaluated in all patients who had treatment with curative intent and a minimum follow-up of six months [5]. Patients who were inoperable and treated with denosumab were excluded from survival analysis.

Data were collected from a secure database on site of disease, child-bearing status, pregnancy, comorbid conditions, surgical management and date of local recurrence. Childbearing was defined according to the published annual conception rate of the Office for National Statistics as any woman between 15 and 44 years of age [6]. Pregnancy was evaluated at the time of diagnosis and included a current pregnancy or a pregnancy within the last six months, including a termination, miscarriage or stillbirth.

Data were analysed with R statistical software (R Foundation for Statistical Computing, Vienna, Austria). The Fisher exact test was used to compare categorical variables and the Wilcoxon rank test to compare numerical variables. Kaplan-Meier survival analysis and the logrank test were used to evaluate differences in local recurrence rate of gender, childbearing status and comorbid conditions [7]. Other factors including site of disease and surgical management were also compared using the logrank test. Cox proportional hazard regression analysis was also performed to analyse confounding variables.

## Results

In total, 195 patients (99 females and 96 males) were identified from the two centres.

The median age was 34 years (interquartile range: 26-48.5 years). Males were significantly older than females (Wilcoxon rank test, p = 0.046).

There were 72 females of childbearing age, of whom 15 were pregnant or had been pregnant in the last six months (21%) at the time of diagnosis. This is a statistically significant difference compared with the highest national conception rate of 8.4%, (Fisher test, p = 0.033) [6].

Although most patients were otherwise fit and healthy, there were 20 patients who had a significant comorbid disease of neoplastic or autoimmune origin (Table 1).

**Table 1:** Clinical comorbidities in 20 out of 195 patients.

Comorbidity	n	
Immune diseases	14	
Inflammatory joint disease	5	Rheumatoid arthritis (3), seronegative arthritis (1), systemic lupus erythematosus (1)
Inflammatory bowel disease	3	Crohn’s disease (1), coeliac (1), ulcerative colitis (1)
Thyroid disease	3	Hyperthyroid (1), hypothyroid (2)
Type 1 diabetes mellitus	2	
Psoriasis	1	
Neoplastic diseases	6	
Benign	1	Prolactinoma (1)
Carcinoma	3	Breast (1), lung (1), squamous cell carcinoma (1)
Lymphoproliferative	2	Acute myeloid leukaemia (1), Hodgkin’s lymphoma (1)

Females were more likely to have a significant comorbidity and had a higher rate of immune disease than males (Fisher test, p = 0.010) (Table 2).

**Table 2:** Comorbidities grouped by gender. The p value refers to the Fisher exact test comparing females and males. Note: the p-value refers to the Fisher exact test comparing females and males.

	Female -	Female +	Male -	Male +	p-Value
Immune disease	87	12	94	2	0.010
Neoplastic disease	94	5	95	1	0.212

Previous international studies into the epidemiology of autoimmune diseases report the prevalence of various autoimmune diseases to range from 5 per 100,000 to 500 per 100,000 [8]. Our study showed a significantly higher prevalence of autoimmune disease in patients with GCTB than an assumed prevalence of 1000 per 100,000 (Fisher test, p = 0.024).

The femur was the most common site of disease (57 cases, 34%). There were 40 cases (24%) in the tibia and 16 (10%) in the distal radius. Intralesional surgical management was performed in 121 (72%), excision in 44 (26%) and amputation in 3 (2%) cases.

There were nine patients who had inoperable disease and were treated with denosumab. A further 18 patients had a very short or no follow-up information available. These 27 patients were excluded from further analysis. Survival analysis was performed on the remaining 168 patients who were treated with curative intent and had a minimum follow-up of six months (Table 3).

**Table 3:** Demographic data of 168 patients who had treatment with curative intent and a minimum follow-up of six months. IQR, interquartile range

	n	Proportion (%)
Male	80	48
Female	88	52
Females of childbearing age	66	75
Pregnancy	13	20
Local recurrence	45	27
	Median (years)	IQR (years)
Age	33	26–47
Follow-up	3.2	2.1–4.7

**Figure 1:**
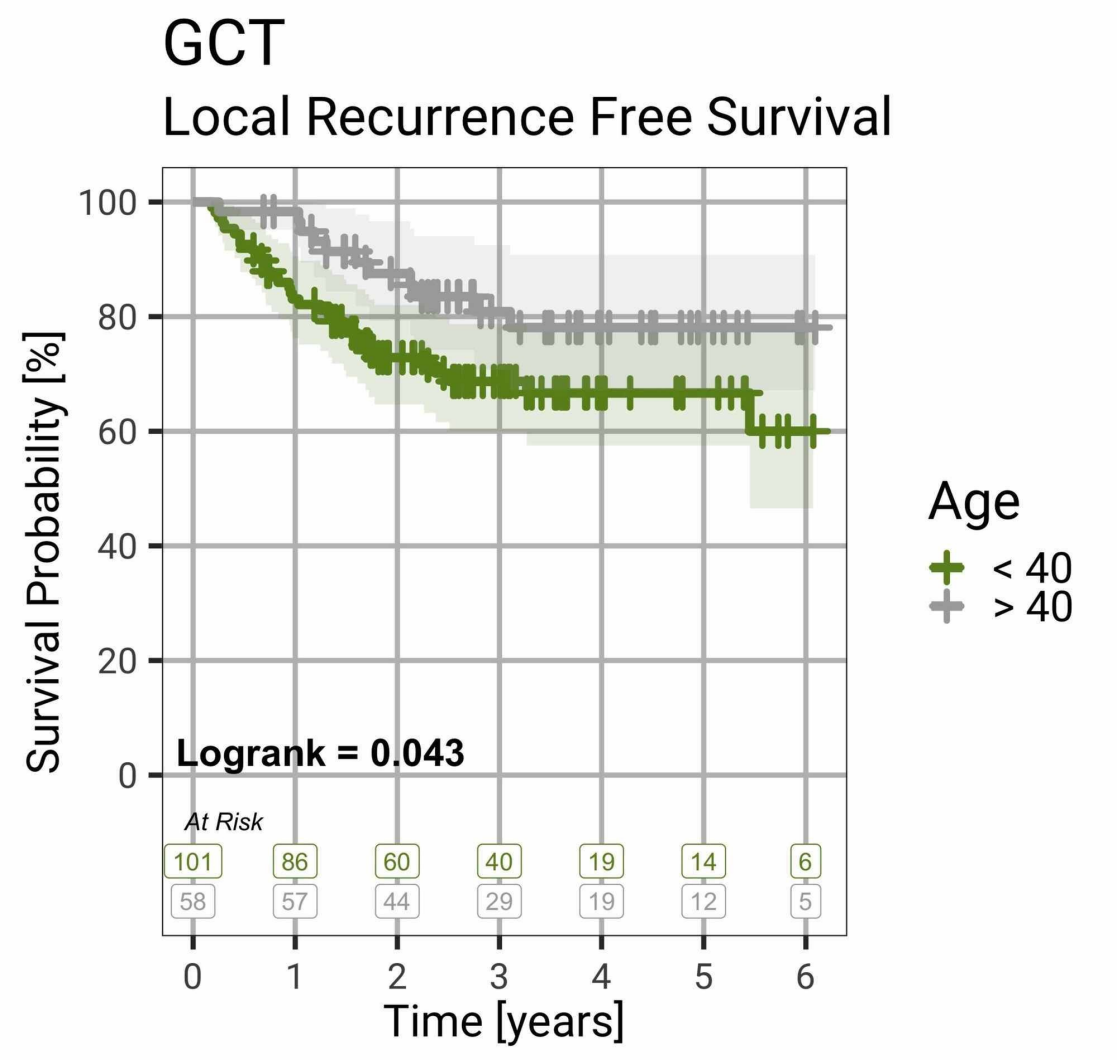
Local Recurrence-Free Survival by Age Group Survival curve showing that patients older than 40 years have less risk of local recurrence than younger patients with GCT of bone (logrank test, p = 0.043). Shaded colours indicate the 95% confidence interval. GCT, giant cell tumour

Figure 1 shows that patients over 40 years of age were significantly less likely to have local recurrence than younger patients (logrank test, p = 0.043).

**Figure 2:**
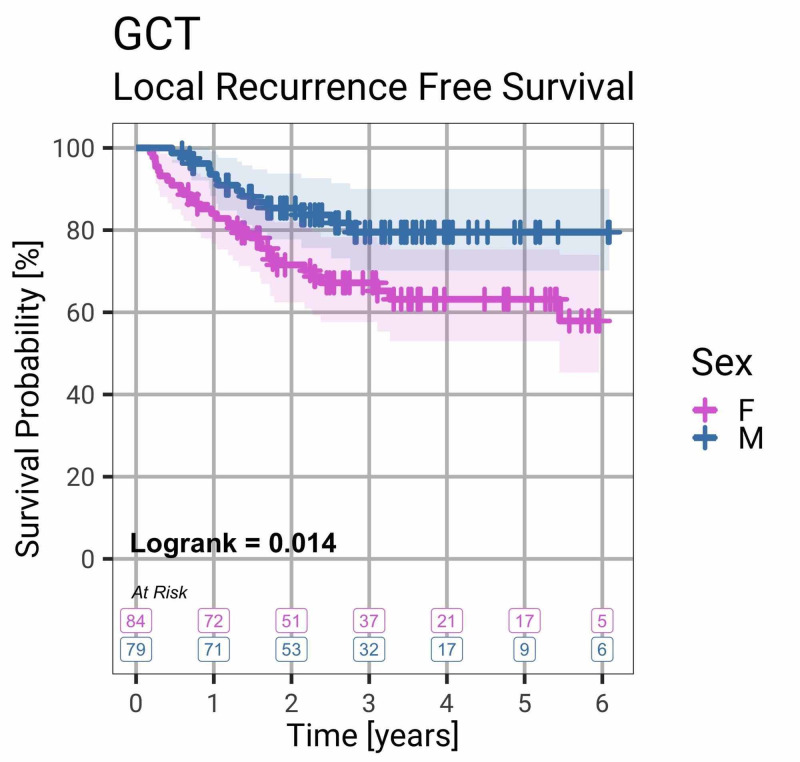
Local Recurrence-Free Survival by Gender Survival curve showing that females are significantly more likely to have local recurrence than males with GCT of bone (logrank, p = 0.014). Shaded colours indicate the 95% confidence interval. GCT, giant cell tumour

Females were more likely to have local recurrence than males (31 females, 13 males; Fisher test, p = 0.008; logrank test, p = 0.014) (Figure 2).

However, pregnant females had a similar local recurrence rate than non-pregnant females (logrank test, p = 0.428). The presence of comorbid conditions did not increase the rate of local recurrence in females (logrank test, p = 0.066).

Of patients with comorbidities, there was no difference in local recurrence rate between the different disease subgroups.

Campanacci grade showed no significant difference in local recurrence rate. However, Campanacci grade 2 with fracture and grade 3 tumours received more aggressive treatment (Table 4).

**Table 4:** Type of treatment received according to Campanacci grade. Note: Higher grade tumours received more aggressive treatment. 2# refers to Campanacci grade 2 with fracture.

Campanacci grade	Amputation	Excision	Intralesional
1	0	1	15
2	0	12	62
2#	0	8	15
3	3	23	29

Campanacci grade 3 tumours that were managed with intralesional treatment were more likely to have local recurrence compared with other Campanacci grades (Table 5).

**Table 5:** Recurrence rate in patients who had intralesional treatment grouped by Campanacci grade. Note: 2# refers to Campanacci grade 2 with fracture.

Campanacci grade	Intralesional treatment	Recurrence (n)	Recurrence (%)
1	15	3	20
2	62	17	27
2#	15	5	33
3	29	15	52

Patients who had excisional surgical management were significantly less likely to have local recurrence (logrank test, p = 0.008).

Of the 168 patients, 34 (20%) had adjuvant preoperative treatment with denosumab (Table 6). The table shows that patients with higher Campanacci grades were more likely to receive treatment with denosumab. However, there was no association between denosumab treatment and the type of surgical treatment received. Patients who had adjuvant treatment with denosumab had no significant difference in local recurrence compared with patients who had surgical treatment only (logrank test, p = 0.145).

**Table 6:** Details of patients who had adjuvant treatment with denosumab. Note: 2# refers to Campanacci grade 2 with fracture.

	n	%	Local Recurrence (n)	Local Recurrence (%)
All	168			
Denosumab	34	20	12	35
No denosumab	134	80	33	25
Campanacci grade				
1: denosumab	0	0	0	0
1: no denosumab	16	100	3	19
2: denosumab	7	9	3	43
2: no denosumab	67	91	16	24
2#: denosumab	4	17	2	50
2#: no denosumab	19	83	3	16
3: denosumab	23	42	7	30
3: no denosumab	32	58	11	34
Surgical treatment				
Amputation: denosumab	1	33	0	0
Amputation: no denosumab	2	67	1	50
Excision: denosumab	11	25	1	9
Excision: no denosumab	33	75	3	9
Intralesional: denosumab	22	18	11	50
Intralesional: no denosumab	99	82	29	24

**Figure 3:**
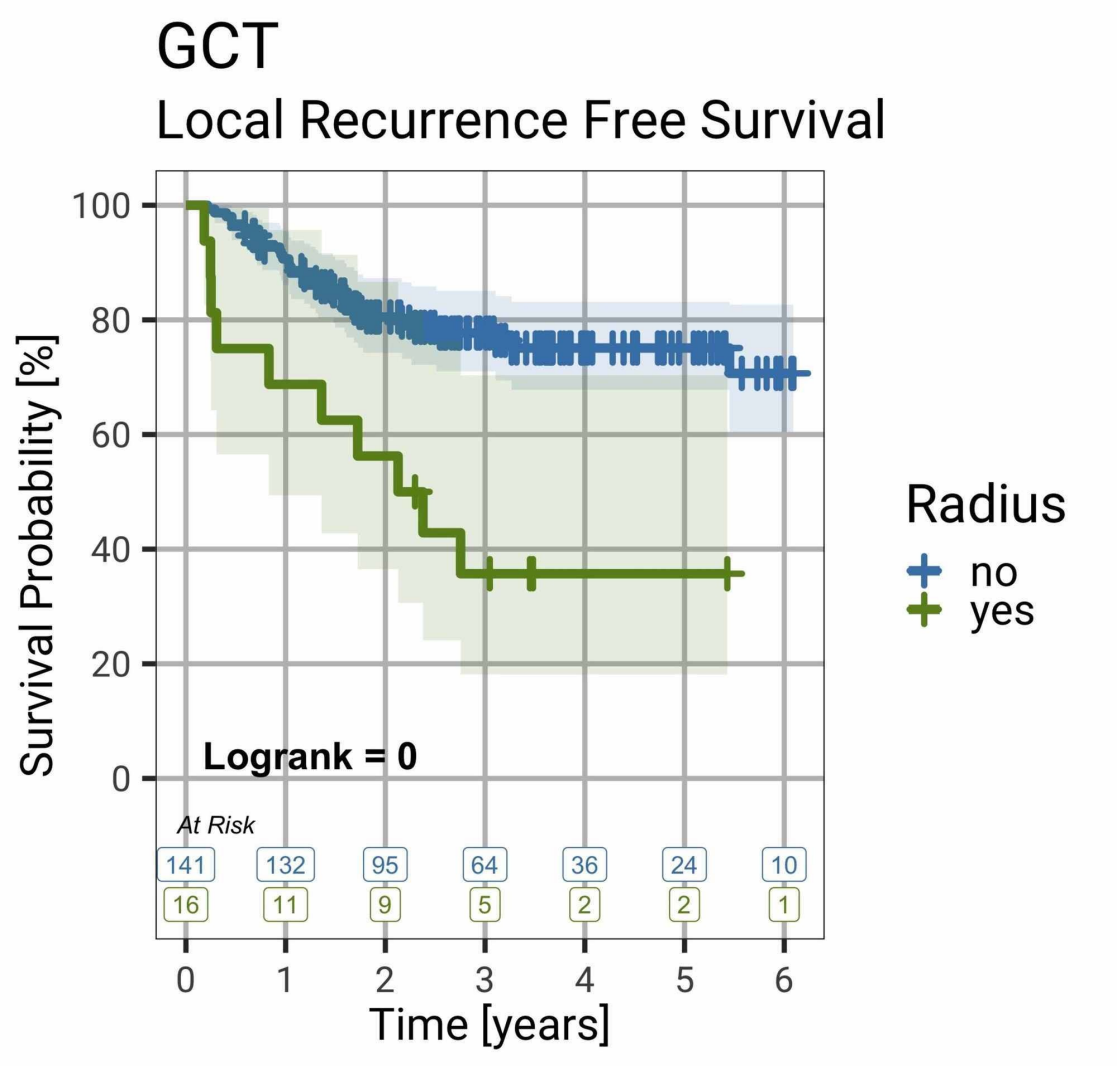
Local Recurrence-Free Survival in GCT of Bone by Site Survival curve showing that patients with GCT of bone in the distal radius are significantly more likely to develop local recurrence (logrank, p < 0.001). Shaded colours indicate 95% confidence intervals. GCT, giant cell tumour

Local recurrence is more likely when the disease is in the distal radius (logrank test, p < 0.001) (Figure 3).

However, of the 16 patients with a distal radial tumour, 11 had intralesional treatment.

Cox proportional hazards regression confirmed that pregnancy (p = 0.573), comorbidities (p = 0.262), pathological fracture (p = 0.896), denosumab treatment (p = 0.093), Campanacci grade (0.383 < p < 0.708 for the different grades) and surgical treatment (p = 0.998) are not associated with a higher recurrence rate. However, tumours in the distal radius do have a significantly higher risk of local recurrence (p < 0.001).

## Discussion

GCTB occurring in mothers has been reported since the 1950s [3]. However, there have been no large studies investigating the relationship between pregnancy and GCTB, or the local recurrence rates of these patients. The existing literature on GCTB relating to pregnancy only contains case reports [2,3,9-13]. Our study evaluates the relationship between pregnancy, GCTB and the risk of local recurrence. The pregnancy rate in patients with GCTB is significantly higher than the national average. It is unlikely that the relationship between pregnancy and GCTB is purely coincidental, but further research is required to establish the underlying mechanism. Although female patients were significantly more likely to have local recurrence than males, pregnancy itself does not increase the risk of local recurrence.

There are three predominant hypotheses for the relationship between GCTB and pregnancy. Firstly, it has been hypothesised that the growth of GCTB in pregnancy is hormonally driven by the changing levels of oestrogen and progesterone in the perinatal period [13]. Some authors have reported the presence of oestrogen receptors on osteoclast-like cells in GCTB [14]. However, when investigating the presence of oestrogen and progesterone receptors in GCTB, oestrogen receptors are found in only approximately 51% of cases of GCTB, and there is no consensus as to the effect of the presence of oestrogen receptors on the biology or clinical behaviour of GCTB [14]. Secondly, it has been hypothesised that there is an immunogenetic relationship between GCTB and pregnancy. If antigens present on the tumour cells are like foetal antigens, maternal immune suppression during pregnancy allows progressive tumour growth [13]. Thirdly, one study found that pregnancy itself causes epigenetic changes due to the interaction between oestrogen, progesterone and histones [15].

Epigenetic changes in the H3.3 histone are well documented to be the sole contributor to the tumorigenesis of GCTB [16]. Therefore, epigenetic changes in the H3.3 histone and pregnancy could be associated with GCTB tumorigenesis [17]. Other authors believe the relationship between GCTB and pregnancy is coincidental as the peak prevalence of GCTB coincides with childbearing years [1]. Epigenetics and histone modifications also contribute to the development of autoimmune diseases including rheumatoid arthritis and systemic lupus erythematosus [18]. However, there are several histone modifications with complex functions that remain poorly understood. Autoimmune diseases are well documented to be associated with an increased malignancy rate due to chronic inflammation increasing cell proliferation, mutagenesis, oncogene activation and angiogenesis [4].

Our study evaluated the relationship between comorbid conditions, in particular those of autoimmune origin, and the relationship between GCTB and local recurrence rates. Females were more likely to have a comorbid condition than males. However, the presence of a comorbid condition did not increase the rates of local recurrence. There was a higher rate of autoimmune conditions in our study group than in the international epidemiological studies. It could be argued that this study is underpowered to compare the prevalence of autoimmune diseases. However, it is challenging to recruit an even larger study group for such a rare tumour.

There are other factors which are more established in the literature that affect rates of local recurrence in GCTB. These include location of the tumour, surgical management and Campanacci grade. Previous studies have shown that local recurrence rates of GCTB located in the distal radius can range from 25% to 88% [19]. This may be attributed to inadequate exposure and dissection of the surgical site due to local anatomical characteristics [20]. Whilst an en bloc excision of the tumour may decrease local recurrence rates, these procedures are more likely to have significant morbidity or complications such as carpus dislocation and deep infection [21]. Our study agrees with the current literature, and patients with GCTB in the distal radius are significantly more likely to develop local recurrence.

Although both centres have a similar treatment strategy for patients with GCTB, variation between surgeons and centres is a limitation of this study. Generally, intralesional treatment with thorough curettage, subsequent burring down of the cavity with a high-speed burr and pulsatile lavage was favoured. Particularly around the knee, the cavity was usually augmented with polymethylmethacrylate cement, but no other adjuvants were used.

In keeping with other published literature, our study demonstrated that intralesional treatment had significantly higher rates of recurrence than excision. However, the increased recurrence rate is felt to be justified to maximise functional outcomes, and therefore intralesional treatment of GCTB is the preferred first-line surgical management for most cases of GCTB [19,21]. The high local recurrence rate in distal radius tumour perhaps would justify a more aggressive approach in this location.

There is conflicting evidence around the association between Campanacci grade and local recurrence rates. Some studies report grade 3 disease to have high recurrence rates, but recently there have been studies reporting lower recurrence rates for grade 3 disease [22]. The lower rates of local recurrence are likely to be because of increased rates of excisional procedures in these patients. Like recent studies, we found that there is no significant difference between the Campanacci grade of disease and local recurrence rates. However, intralesional treatment of Campanacci grade 3 tumours was associated with a higher recurrence rate than the use of intralesional treatment in other Campanacci grades.

## Conclusions

There are a variety of factors that affect local recurrence in GCTB. Disease in the distal radius, intralesional surgical management, being female and age younger than 40 years all increase the risk of local recurrence. Although there are higher rates of pregnancy and comorbidities in patients with GCTB, this does not appear to affect the local recurrence rate.

Further studies involving multiple centres are required for confirmation of the relation between comorbid conditions, pregnancy and GCTB. Furthermore, a better understanding of the underlying biological pathways is required.
